# Spinal cord ischemia following pediatric cardiac arrest: An unexplored complication of hypoxic ischemic injury

**DOI:** 10.1016/j.jocn.2026.111916

**Published:** 2026-02-13

**Authors:** Morgann Loaec, Arastoo Vossough, Conall Francoeur, Kathryn Graham, Ryan W. Morgan, Alexis A. Topjian, Matthew P. Kirschen

**Affiliations:** a Department of Anesthesiology and Critical Care Medicine, Perelman School of Medicine at the University of Pennsylvania, Children’s Hospital of Philadelphia, Philadlephia, PA, USA; b Department of Radiology, Perelman School of Medicine at the University of Pennsylvania, Children’s Hospital of Philadelphia, Philadlephia, PA, USA; c Department of Pediatrics, Division of Pediatric Critical Care Medicine, McGill University Health Centre, Montreal Children’s Hospital, Montreal, Canada

**Keywords:** Hypoxic-ischemic spinal cord injury, Pediatric cardiac arrest, Neuroimaging, Neuroprognostication

## Abstract

**Background:**

Hypoxic-ischemic brain injury (HIBI) is a well-described sequela of pediatric cardiac arrest, but the epidemiology and clinical implications of hypoxic-ischemic spinal cord injury (HISCI) remain poorly understood. Only isolated reports describe HISCI following cardiopulmonary resuscitation (CPR). We aimed to describe the incidence, imaging characteristics, and clinical context of HISCI in pediatric cardiac arrest patients undergoing clinically indicated MRI.

**Methods:**

We conducted a single-center retrospective descriptive case series of consecutively identified pediatric cardiac arrest patients who underwent spinal magnetic resonance imaging (MRI) within two weeks of resuscitation (2018–2023). Cases were identified from an institutional cardiac arrest database. MRI scans were independently reviewed by a pediatric neuroradiologist for evidence of HISCI.

**Results:**

Of 717 cardiac arrest patients, 36 (5%) underwent spinal MRI within two weeks of arrest, primarily for trauma evaluation (72%). Four patients (11%) had MRI evidence of HISCI. All four experienced out-of-hospital cardiac arrest with CPR durations ranging from 8 to 90 min and initial serum lactate > 4 mmol/L. Two arrests were traumatic. All four patients had concomitant HIBI, and two met criteria for death by neurologic criteria. Among the 32 patients without HISCI, 9 (28%) had HIBI and 19 (59%) had traumatic arrest.

**Conclusions:**

HISCI was identified in 11% of pediatric cardiac arrest patients who underwent post-arrest spinal MRI for clinical indications. Recognition of HISCI has potential implications for neuroprognostication, rehabilitation planning, and determination of brain death by neurologic criteria. Larger prospective studies are needed to define the incidence, risk factors, and outcomes of HISCI following pediatric cardiac arrest.

## 1. Introduction

Hypoxic-ischemic brain injury (HIBI) has been well described after pediatric cardiac arrest.[1] Hypoxic-ischemic spinal cord injury (HISCI) has been identified in some patients following cardiac arrest, however, its prevalence is unknown and it is unclear whether the spinal cord has the same susceptibility to hypoxic-ischemic injury as the brain.[2,3] Though it is less well characterized, HISCI has implications for both neuro-prognostication and rehabilitation following cardiac arrest, which may be considered in addition to the prognostic implications of HIBI. Importantly, spinal cord injury may contribute to persistent motor deficits, autonomic dysfunction, and functional impairment that are not explained by cerebral imaging alone, complicating prognostication and counseling for families.[4].

A recent case series and literature review highlighted post-cardiac arrest HISCI in both children and adults.[5] Among the 81 adults and 9 children with HISCI, ischemia was most commonly identified at cervical and thoracic levels, although multi-level ischemia and entire cord involvement was reported. Ischemia occurred without clear risk factors, was often discordant with the severity of HIBI, and the degree of functional impairment varied. However, existing reports are largely derived from selectively imaged or published cases, limiting interpretation regarding incidence, clinical context, and spectrum of disease.

Given our limited understanding of the epidemiology of post-cardiac arrest HISCI and our limited understanding of the implications of HISCI, we sought to characterize spinal cord ischemia in a cohort of children from a large quaternary care center within an existing cardiac arrest database. The primary aim of this study was to describe the occurrence, imaging characteristics, and clinical context of HISCI among pediatric cardiac arrest patients who underwent clinically indicated spinal magnetic resonance imaging using a consecutive descriptive case series to provide hypothesis-generating data that may inform future prospective studies.

## 2. Methods

This was a single center retrospective descriptive case series performed at a quaternary pediatric center. The Children’s Hospital of Philadelphia Institutional Review Board determined this study exempt from consent for human research (IRB 23–021700). Using an institutional cardiac arrest database, we identified children who were resuscitated from an in-hospital (IHCA) or out-of-hospital cardiac arrest (OHCA) between 2018–2023 as the source population. Patients met inclusion criteria if they had a cervical, thoracic, lumbar, or whole spine magnetic resonance imaging (MRI) scan performed for clinical indications within two weeks of their arrest.[6] There were no exclusion criteria. MRI images were independently reviewed by a pediatric neuroradiologist (AV) to assess for the presence of HISCI and other potential confounding findings. Demographic information, arrest characteristics, and relevant laboratory data were abstracted from an institutional cardiac arrest database. Manual chart review was conducted to determine the reason spinal MRI scans were obtained. Categorical variables were reported as counts and percentages, and continuous variables as medians with interquartile ranges (IQR). Secondary to the rare nature of HISCI and low incidence expected within our cohort, the purpose of this analysis was case characterization and no hypothesis testing was planned or performed.

## 3. Results

Five percent (36/717) of patients in our institutional cardiac arrest database underwent spinal MR imaging within two weeks of cardiac arrest. Scans were primarily completed as part of a trauma evaluation (72%) or to further evaluate for a suspected neurologic condition that could have contributed to the cardiac arrest including in patients with a pre-existing central nervous system malignancy, cervical spinal stenosis, or Chiari malformation (25%). One patient underwent routine imaging in the process of planning for vertical expandable prosthetic titanium rib (VEPTR) surgery following a brief IHCA (3%). The spinal imaging in this cohort included sagittal and axial T1-weighted, sagittal and axial T2-weighted, and when available, multi-shot readout segmented diffusion weighted imaging.

Of the 32 patients who had a spinal MRI without HISCI, the median CPR duration was 6 [IQR 2–10] minutes, initial serum lactate was 3.4 [1.7–4.9] mmol/L, and nine had HIBI. Nineteen patients (59%) had confirmed trauma. Nineteen patients (59%) had at least mild ligamentous injury, only one patient had evidence of ligamentous injury in the absence of other traumatic injury.

**Fig. 1.**
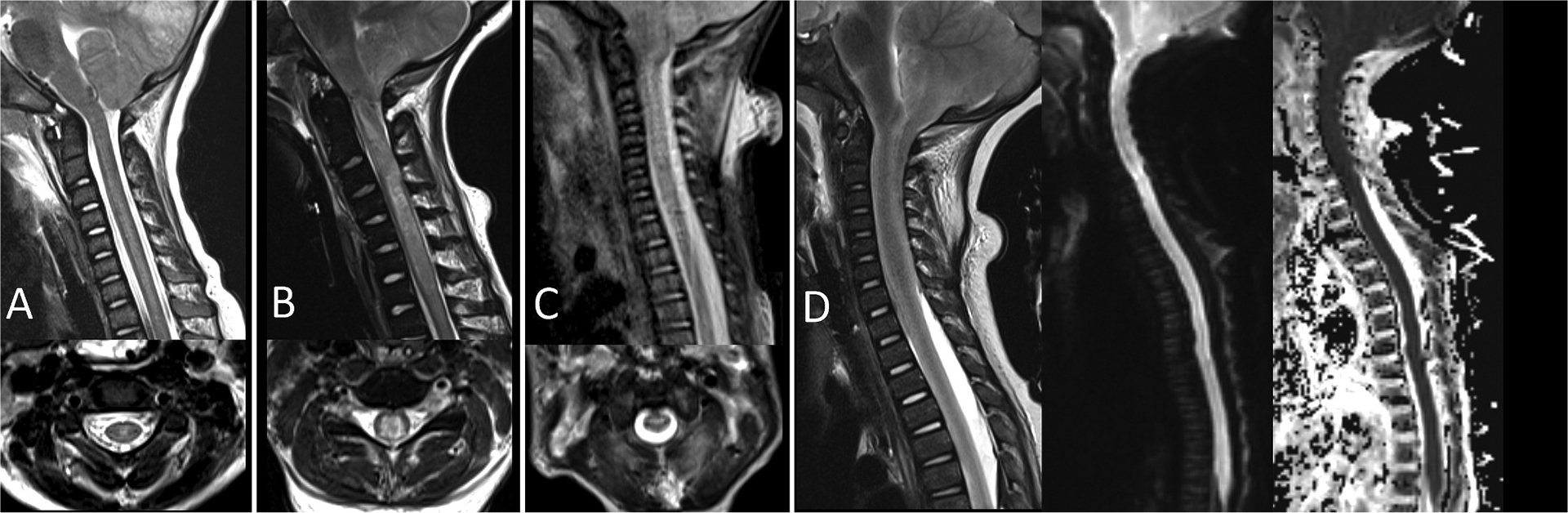
Various degrees of spinal cord ischemic injury in three patients after cardiac arrest. A. A 5-year-old patient after cardiac arrest. Sagittal and axial T2-weighted images demonstrate faint hyperintense signal in the central cervical spinal cord gray matter. B. A 7-year-old patient after cardiac arrest. Sagittal and axial T2-weighted images demonstrate tonsillar herniation, marked cervical spinal cord swelling, and marked hyperintense signal in the central spinal cord involving both gray and white matter. C. A 5-month-old patient after cardiac arrest of unknown etiology but suspected to be sudden infant death syndrome. Sagittal and axial T2-weighted images demonstrate HISCI at the level of C1-C3 without evidence of trauma. D. A 10-month-old patient after cardiac arrest and suspicion of non-accidental trauma. Sagittal T2-weighted, sagittal diffusion-weighted, and sagittal ADC (apparent diffusion coefficient) images demonstrate marked tonsillar herniation, with diffuse cord swelling and ischemic injury of the entirety of the spinal cord.

Four patients (11%) had HISCI (Table 1, Fig. 1). All of these patients had an OHCA with CPR duration ranging from 8 to 90 min and initial serum lactate > 4 mmol/L. All four patients had spinal ligamentous injury, two of whom had confirmed trauma. The two patients with traumatic OHCA had additional spinal injuries: one had a spinal epidural hematoma and one had cervical spinal cord contusion. All patients had concomitant moderate (n = 1) or severe (n = 3) HIBI.

*Case 1:* A five-month-old infant presented with OHCA of unknown etiology and suspected sudden unexpected infant death. The total CPR duration was 35 min. This patient received a complete trauma evaluation, without any evidence to suggest trauma as the source of cardiac arrest. A cervical spine MRI was obtained as part of a trauma evaluation. The patient had severe HIBI and HISCI of the cervical cord at the level of C1-C3. The patient died from withdrawal of life-sustaining therapy due to poor neurologic prognosis.

*Case 2:* A ten-month old infant presented after an OHCA secondary to non-accidental trauma. The duration of CPR was unknown. The initial post-ROSC serum lactate was 8 mmol/L. A cervical and thoracic spine MRI was obtained as part of the trauma evaluation. The imaging revealed severe HIBI, tonsillar herniation, spinal cord edema, and ischemia of the entire imaged spinal cord. The patient subsequently underwent an evaluation for brain death/death by neurologic criteria (BD/DNC which included a radionuclide brain perfusion scintigraphy study as an ancillary test due to the known HISCI. The patient’s neurologic exams, apnea tests, and ancillary study were consistent with BD/DNC and the child was declared BD/DNC.

*Case 3:* A five-year-old child presented after an OHCA secondary to an arrhythmia and received 90 min of CPR. The child had a brain computed tomography (CT) scan completed to evaluate for neurologic causes of the arrest which identified an os odontoideum defect. This abnormality was further investigated by a cervical spine MRI. The brain MRI revealed moderate watershed ischemia, and the cervical spine MRI was notable for ligamentous injury and HISCI in the central cervical cord gray matter. This patient was later discharged to inpatient rehabilitation with impairments in strength, coordination, and cognition.

*Case 4:* A 7-year-old child presented following an OHCA due to pedestrian versus motor vehicle collision with traumatic cardiac arrest. CPR duration was 8 min. This patient underwent cervical spine MRI as part of their trauma evaluation. The patient had severe HIBI, tonsillar herniation, and HISCI in the cervical spine. The patient was evaluated for BD/DNC but did not meet clinical criteria due to spontaneous respiration during the apnea test. This patient was discharged with significant neurologic deficits and technology dependence.

## 4. Discussion

In this single-center retrospective case series, 11% of patients with spinal imaging performed following cardiac arrest for clinical indications had evidence of HISCI. Within a large cardiac arrest database, this was 0.5% of the total population of patients managed for post-arrest care, however no standard post-arrest spinal imaging protocol existed and only 36 of 717 patients had a spinal MRI performed. HISCI was present in patients with and without known trauma. Although all patients with spinal ischemia had at least moderate HIBI, ten patients with severe HIBI did not have detectable HISCI by MRI. Given the limited size of our cohort, we could not determine associations between patient and cardiac arrest characteristics and HISCI.

This case series further supports that children may be at risk for HISCI following cardiac arrest. Francoeur et al. recently published the first pediatric case series and rapid literature review describing HISCI after resuscitated cardiac arrest, underscoring the limited data available on this entity. Their five consecutive cases all involved prolonged cardiac arrest with HIBI, and spinal MRI was performed because of concerning clinical findings such as absent rectal tone or focal deficits, reflecting a growing institutional awareness of this condition.[5] Our findings extend those of Francoeur et al. by estimating the frequency of HISCI among children who underwent post-arrest spinal MRI over a five-year period at a large quaternary center. In contrast to their study, spinal imaging in our cohort was performed primarily for trauma assessment or to evaluate other suspected neurologic conditions, rather than specifically to investigate spinal ischemia. The indication for spinal imaging was most often to assess for traumatic spinal cord injury which may introduce a multifactorial source of HISCI including hypoperfusion, reperfusion injury, and traumatic injury. Moreover, imaging was often limited to the cervical spine, which may have led to under-recognition of ischemia in thoracic or lumbar regions. Despite these differences, both series included patients who required ancillary nuclear perfusion imaging to establish BD/DNC, highlighting how HISCI may confound this examination in post arrest patients. Notably, our series also included two patients who survived to hospital discharge requiring inpatient rehabilitation for motor and coordination deficits, a finding not described in the Francoeur series. This additional case information highlights the possible implications of HISCI among cardiac arrest survivors. Taken together, these findings suggest that spinal cord ischemia after cardiac arrest may be more common than previously recognized and underappreciated with our current imaging approaches.

Detecting HISCI has important implications for ICU management, rehabilitation candidacy, and prognostication. HISCI may impair muscle strength, coordination, or respiratory mechanics, which may confound the neurologic exam and impact neuroprognostication following cardiac arrest.[4] Decisions regarding withdrawal of life-sustaining therapies after cardiac arrest are often driven by the severity of HIBI. In some situations, there is discordance between biomarkers of brain injury like neuroimaging, and the patient’s neurologic exam. This may be due in part to under recognition of HISCI since spine imaging is not incorporated into most post-arrest neuroimaging protocols.[7–10] For example, one patient in our series survived to discharge without severe HIBI and required inpatient rehabilitation. Recognition of HISCI could improve rehabilitation planning by enabling the team to individualize therapy for spinal cord deficits while also addressing coexisting brain injury and critical illness neuromyopathy. Knowledge of a post-arrest patient’s spinal cord injury may complement the prognostic value of existing brain imaging protocols post-arrest. The mechanisms underlying the selective vulnerability of spinal versus cerebral tissue to hypoxic-ischemic injury remain uncertain. Characterization of the epidemiology of HISCI and the association between the burden of ischemia on brain and spinal imaging post-arrest will require a large prospective observational study.

HISCI may impair accurate interpretation of motor responses and respiratory function during evaluation for BD/DNC. As a result, the presence of a HISCI requires ancillary testing to make a BD/DNC determination, assuming the remainder of the neurologic exam and apnea test are consistent with BD/DNC.[11,12] In epidemiological studies about BD/DNC, the presence of HISCI has not consistently been reported. Understanding the prevalence and risk factors for HISCI following cardiac arrest may help to inform imaging practices prior to evaluating patients for BD/DNC.

Detection of hypoxic-ischemic spinal cord injury can be challenging on conventional MRI, particularly with diffusion imaging sequences. Diffusion imaging is classically obtained as single-shot echoplanar imaging and is limited due to encasement of the spinal cord in the osseous vertebral canal and proximity to air in the lung (thoracic segments), leading to susceptibility artifacts, signal loss, and geometric distortion, often precluding accurate diagnosis. Widespread usage of parallel imaging with echoplanar diffusion has somewhat improved image quality, but challenges remain. Use of newer and more advanced diffusion sequences with decreased geometric distortion and susceptibility can increase the detectability of acute spinal cord ischemia.[13] These can include multi-shot echoplanar imaging preferably with readout segmentation, non-echoplanar imaging, or parallel transmit technology with dynamic excitation pulses to achieve a selective reduced field-of-view with decreased distortion. All these techniques have shown to considerably improve spinal diffusion imaging, but they may not be as widely available or underutilized. Prospective observational studies involving appropriate imaging technique and neuroradiology review are needed to further characterize the prevalence of spinal cord ischemia following pediatric cardiac arrest.

This study has several limitations. First, this was a single-center retrospective case series and cannot assess prevalence or the full spectrum of HISCI after pediatric cardiac arrest. Imaging was obtained for a clinical suspicion of injury and thus may overestimate or underestimate the prevalence of HISCI post arrest. This case series brings attention to a rare and under-reported complication of pediatric cardiac arrest, but future prospective studies are needed to characterize prevalence and test for associations. Second, MRI imaging was largely limited to the cervical spine for trauma assessment. Future studies that include thoracic and lumbar imaging may better capture the extent and severity of HISCI, since watershed regions of the spinal cord, especially the upper thoracic and thoracolumbar segments, may be more susceptible to ischemia during periods of global hypoperfusion such as cardiac arrest. The images used in this case-series are from clinically indicated spinal MRI and may under-represent the true incidence of HISCI due to the unimaged portions of the spine. This selective imaging also introduces potential selection bias and limits generalizability. Third, conventional MRI sequences may have limited sensitivity for detecting acute ischemia. Fourth, spinal images were reviewed by one pediatric neuroradiologist and we did not assess inter-rater reliability for the diagnosis of HISCI and we did not assess inter-rater reliability for the diagnosis of HISCI. Fifth, clinical characterization of HISCI related deficits was limited, as most patients had concurrent HIBI or other traumatic injuries, making it difficult to separate the contribution of spinal cord injury to overall outcomes. Finally, the small sample size precludes meaningful statistical analysis of risk factors or outcomes and underscores the need for larger, prospective studies.

## 5. Conclusion

This single-center consecutive case series demonstrates the presence of HISCI in children who had a clinically indicated spine MRI performed following OHCA. Recognition of HISCI may have implications for neuroprognostication, rehabilitation candidacy, and BD/DNC evaluation following cardiac arrest. Larger prospective studies are needed to define its true incidence, risk factors, and long-term outcomes.

## Figures and Tables

**Table 1:** Patients with spinal cord ischemia following pediatric cardiac arrest.

Case	Age (years)	Sex	Arrest Location	CPR Duration (minutes)	Initial Post-Arrest Lactate (mmol/L)	Cause of Arrest	Confirmed Trauma	Indication for Spine MRI	Spinal Cord Ischemia	Ligamentous Spine Injury	Hypoxic-Ischemic Brain Injury	Patient Outcome
1	0.4	M	OHCA	35	6.7	Unknown (suspected sudden infant death syndrome)	No	Trauma evaluation	C1-C3	Moderate	Severe	Death
2	0.8	F	OHCA	unknown	8	Non-accidental trauma	Yes	Trauma evaluation	Entire Cord	Moderate	Severe	Death
3	5	F	OHCA	90	9.6	Arrythmia	No	Follow up for Os odontoideum identified on CT scan	Cervical Cord	Mild	Moderate watershed	Survived
4	7	F	OHCA	8	4.2	Trauma	Yes	Trauma evaluation	Cervical Cord	Mild	Severe	Survived

OHCA (out of hospital cardiac arrest).
